# The Polish adaptation of the measurements of rule-governed behaviors: Generalized Pliance Questionnaire, Generalized Tracking Questionnaire and Generalized Self-Pliance Questionnaire

**DOI:** 10.1371/journal.pone.0283795

**Published:** 2023-04-05

**Authors:** Joanna Dudek, Maria Cyniak-Cieciura, Paweł Ostaszewski

**Affiliations:** 1 Faculty of Psychology in Warsaw, Center for Behavioral Research in Decision Making, SWPS University, Warsaw, Poland; 2 Advanced Clinical Studies and Therapy Excellence Center, Institute of Psychology, SWPS University, Warsaw, Poland; 3 Center for Behavioral Research in Decision Making, Institute of Psychology, SWPS University, Warsaw, Poland; Universita degli Studi Europea di Roma, ITALY

## Abstract

In some circumstances rule-governed behavior, a behavior that is governed by verbal rules instead of environmental consequences, may be beneficial for human beings. At the same time, rigid rule following is associated with psychopathology. Thus measurement of rule-governed behavior may be of special use in a clinical setting. The aim of this paper is to assess the psychometric properties of Polish adaptations of three questionnaires measuring generalized tendency to engage in various types of rule-governed behaviors: Generalized Pliance Questionnaire (GPQ), Generalized Self-Pliance Questionnaire (GSPQ), Generalized Tracking Questionnaire (GTQ). A forward-backward method was used for translation. Data was collected from two samples: general population (N = 669) and university students (N = 451). To measure the validity of the adapted scales the participants filled in a set of self-assessed questionnaires: Satisfaction with Life Scale (SWLS), Depression, Anxiety, and Stress Scale– 21 (DASS-21), General Self-Efficacy Scale (GSES), Acceptance and Action Questionnaire–II (AAQ-II), Cognitive Fusion Questionnaire (CFQ), Valuing Questionnaire (VQ) and Rumination—Reflection Questionnaire (RRQ). The exploratory and confirmatory analyses confirmed the unidimensional structure of each of the adapted scales. All of those scales presented good reliability (internal consistency measured with Cronbach Alpha) and item-total correlations. The Polish versions of questionnaires presented significant correlations in the expected directions with relevant psychological variables in line with the original studies. The measurement occurred invariant across both samples as well as gender. The results provide evidence that Polish versions of GPQ, GSPQ and GTQ present sufficient validity and reliability to be used in the Polish-speaking population.

## Introduction

To provide a behavior-analytic account of complex human behavior such as thinking and problem solving, in 1966 Skinner [1] introduced the term rule-governed behavior (RGB), defined as behavior that is under the control of rules and instructions, in contrast to contingency-shaped behaviors which are under control of direct contingencies in the environment. The functional behavioral analysis of the concept was first proposed almost 20 years later by Zettle and Hayes [2] and elaborated in detail within the framework of the Relational Frame Theory [3].

Beginning with Skinner, researchers emphasize that the ability to generate and follow verbal rules may be beneficial especially in contexts where learning through direct experience is dangerous (e.g. look both ways before crossing the street) or contingencies are delayed (e.g. attend classes and study to get the diploma). Thus, RGB helps people to achieve goals, learn from the experience of others, and cope with events before they occur [4]. However, under certain circumstances, RGB can also produce undesired consequences, such as insensitivity to real environmental contingencies, rigidly following verbal rules despite their effectiveness or even harmful outcome of rule following, or persistent avoidance [5–7].

Therefore, the concept of RGB has become particularly important in the domain of clinical behavior analysis, as it provides both explanation of the development of a number of psychopathological symptoms [5–8], and helps to develop psychotherapeutic interventions, such as Acceptance and Commitment Therapy (ACT) with its focus on the psychological flexibility model [9]. To support both basic and clinical research on the RGB, reliable and valid methods of assessing the behaviors are required. The aim of the present paper is to present the validation process of the three recently developed questionnaires measuring two functional types of RGB, generalized pliance (and self-pliance) and generalized tracking [10–12] in a Polish population.

Different functional types of RGB were first introduced by Zettle and Hayes [2], who distinguished pliance and tracking as two most fundamental functional classes of RGB, and a third type, augmenting, operating together with the two former classes by verbally changing the reinforcing or punishing strength of consequences included in the rules.

Pliance is defined as a functional class of rule-governed behavior under the control of history of multiple interactions in which the speaker provides the listener with the reinforcement contingent on the correspondence between the rule (e.g. do not touch hot pot) and the relevant behavior (refraining from touching the pot). An example of reinforcement in such circumstances may be praising the individual (e.g. great that you did not touch the pot [2, 3, 13]). Taking into account that the listener and the speaker may be the same person [14], under some circumstances those rules can also be generated by the individual and then called self-rules [10, 11].

Pliance, being the first type of rule-following developed [13], over-generalizes at some point in the child’s development. Yet, social interactions lead to contextualizing pliance (so that the child can distinguish when it is appropriate) and establishing tracking to help her to recognize natural consequences of her behavior [5, 7].

Lack of such learning history may lead to generalized pliance [11]. Generalized pliance can be problematic when it becomes the main source of impact on human behavior, as socially mediated consequences are less predictable and controllable than other types of consequences which may lead to lower contact with sources of positive reinforcement. Individuals displaying generalized pliance may be particularly insensitive to direct contingencies (e.g. a person believes that in order to obtain social approval she needs to start smoking, so she does that ignoring the negative consequences of smoking). As the child develops and gains fluency in relational framing, plys become more abstract (e.g. a person believes she needs to be a ‘good’ in order to be loved [15]) which may increase the likelihood social approval to be the main source of reinforcement.

As many of social rules treat aversive private events as events that need to be controlled or avoided, individuals displaying generalized pliance are more likely to engage in rigid patterns of behaviors and a tendency to engage in experiential avoidance—attempts to avoid, control or get rid of unwanted internal experiences even when doing so is harmful for the individual [5, 16].

Considering that generalized pliance may lead to losing the contact with the sources of positive reinforcement and behaviors being controlled by negative reinforcement (avoidance) it is considered a risk factor for developing psychopathology (e.g. [5, 6, 8] and psychological inflexibility–difficulties in engaging in meaningful actions due to the presence of unpleasant internal experiences that the person wants to avoid, get rid of or control [4].

In child development, pliance is seen as a condition to develop tracking [13]. In contrast to pliance, tracking is sensitive to the direct environmental contingencies, so that if they change the individual is changing the behavior accordingly [2] and it may be regarded as a flexible rule-governed behavior [12]. Tracking is a functional class of behaviors under the control of the history of multiple exemplars in which following the rule leads to natural consequences derived from the way the world is arranged (e.g. following the rule “when it is cold, wear a warm coat” leads to feeling warm even when it is cold outside [2, 3, 13]). Generalized tracking is a pattern of behaviors that may be developed when an individual has been exposed to multiple interactions in which she has been encouraged to observe and describe functional relationships among events, e.g. recognize natural consequences of her behavior. The individual engaged in tracking, behaving both as speaker and listener, learns to establish functional relationships among events and adjust her behavior accordingly [12]. Despite the interest in rule-governed behaviors especially in the area of contextual behavioral science, there are a number of limitations to experimental research investigating pliance and tracking [17]. One of the proposed explanations involves noting that both pliance and tracking are listener-oriented concepts [18] and therefore they cannot be produced by the speakers as the participants’ personal learning history may influence their performance more than the experimental rules [11, 12].

Thus, researchers proposed alternative strategy of measuring pliance and tracking by developing self-report measures, which explore the perspective of the listener and investigate the individual’s learning history and personal experience with formulating and following rules [11, 12]. A measure of generalized pliance, Generalized Pliance Questionnaire (GPQ, [11]), self-pliance, Generalized Self-Pliance Questionnaire (GSPQ, [10]), and a measure of generalized tracking, Generalized Tracking Questionnaire (GTQ, [12]), were created.

Empirical evidence with the GPQ shows that generalized pliance is connected to various aspects of psychological inflexibility and measures of distress [11]. Higher scores in the GPQ were predictive of lower levels of mindfulness and sensitivity to changing contingencies [19]. Generalized pliance was positively correlated with repetitive negative thinking, dysfunctional attitudes, difficulties in valued living, and negatively correlated with life satisfaction [11].

Generalized tracking measured with GTQ was negatively correlated with generalized pliance, experiential avoidance, tendency to ruminate and emotional symptoms and positively correlated with valued living, life satisfaction and general self-efficacy and a wide range of executive functions [12].

Measuring self-reported patterns of rule-governed behaviors, such as the GPQ and GTQ, may lead to development of research on complex human behavior by broadening the knowledge on rule-governed behaviors, its impact and development (e.g. differences across age, gender, and cultures). These instruments may also be used to explain variability of results in experimental analyses, predict development of psychopathology and behavioral rigidity, and eventually, to analyze mediators and moderators of psychological interventions (e.g. Acceptance and Commitment Therapy). Thus, it seems important to provide researchers and practitioners in the area of clinical behavior analysis and contextual behavioral science with appropriate questionnaires in countries and cultures in which people speak other languages than English or Spanish, such as Polish. The strategy of adopting already existing questionnaires with good psychometric qualities seems a good step, because it allows multilingual and multicultural comparisons of the same phenomena. We hope that the adaptation of GPQ. GSPQ, and GTQ into Polish language will aid in the development of contextual behavioral science in general, allow for multicultural analysis of the RGB, and most of all, provide a growing number of researchers and practitioners in Poland with valid questionnaires measuring RGB, widening the scope of both basic and applied research conducted.

## Materials and methods

### Scales’ adaptation

The original items of the Generalized Pliance Questionnaire (GPQ18–18 items, and its shorter version—GPQ9), Generalized Tracking Questionnaire (GTQ– 11 items), and Generalized Self-Pliance Questionnaire (GSPQ– 12 items) were translated to Polish by three independent translators, who were practitioners in third wave cognitive-behavioral approaches and/or experts in contextual behavioral science. Then, three independent versions of the Polish items were presented to three independent judges–scientists and practitioners in the area of contextual and behavioral science as well as third wave cognitive-behavioral therapy. Final versions of items’ translations were chosen based on the judges’ opinions, back-translated to English and accepted by the author of the original questionnaires. The original instructions and response scales were kept (see [10–12], thus each item was rated on a scale from 1 to 7 (with 7 = *always true* and 1 = *never true*). These versions of the scales were used in the study aiming at verifying their psychometric properties.

### Participants

The psychometric properties of Polish versions of GPQ18, GPQ9, GTQ, and GSPQ were checked in two independent studies and different samples described below.

### Sample A–students’ sample

The participants were recruited via university SONA research panel. Students received credits for taking part in the study in accordance with the university policy. The study was closed after the data from at least 400 participants was collected. The only exclusion criteria were being less than 18 years old. A total of 451 people completed the study: 371 women (82.3%) and 80 men (17.7%) in the age of 18–64 (*M* = 27.61, *SD* = 8.27). Most of them had a secondary educational level (*N* = 245, 54.3%), 206 (40.7%) declared higher education. Most of the participants were living in the city with more than 500 000 residents (*N* = 252, 55.9%) and the least—in the rural area (*N* = 48, 10.6%). A total of 87 people (19.3%) were living in a city with less than 100 000 residents and 64 people (14.2%) in a city with 100–500 000 residents.

### Sample B–a general sample

The participants were recruited by the Pollster research panel (https://pollster.pl/), the only exclusion criteria were being less than 18 years old. The data collection was stopped when data from at least 600 participants was collected. A total of 669 people completed the study: 333 women (49.8%) and 336 men (50.2%) in the age of 18–65 (*M* = 40.96, *SD* = 13.49). Most of them had a secondary educational level (*N* = 360, 53.8%), 272 (40.7%) declared higher education and 37 (5.5%) finished only primary school. Most of the participants were living in the city with less than 100 000 residents (*N* = 254, 38%), 25.7% (*N* = 172)—in the city with 100–500 000 residents, 18.7% (*N* = 125) in the rural area, and 17.6% (*N* = 118) in a city with more than 500 000 residents.

### Procedure

The study was conducted online between March—June 2021. The participants were asked to fill in a set of self-report questionnaires: a short demographic questionnaire, the Polish versions of GPQ18, GTQ and GSPQ and a few other measures aiming at verifying GPQ18, GPQ9, GTQ and GSPQ’s validity (their short description is presented below). All of the participants signed an informed consent. The study was conducted following the Declaration of Helsinki and received a positive opinion from the local Ethics Committee (Nr 5/2021).

### Other measures

#### Satisfaction with Life Scale

The Polish version of the Satisfaction with Life Scale (SWLS [20, 21]) was used to measure self-perceived well-being. It consists of five items. Participants rated each item on a 7-point scale, ranging from 7 = *strongly agree* to 1 = *strongly disagree*. Higher scores indicate a greater level of life satisfaction. Medium to large negative correlations were expected between SWLS and GPQ18, GPQ9, GSPQ, and positive—with GTQ.

#### Depression, Anxiety, and Stress Scale– 21

The Polish version of Depression, Anxiety, and Stress Scale– 21 (DASS-21 [22, 23]) was used to measure the level of depression, anxiety and stress symptoms. It consists of 21 items and a 4-point Likert-type scale (3 = *applied to me very much*, *or most of the time*; 0 = *did not apply to me at all*). It contains three subscales: Depression, Anxiety, and Stress with higher scores indicating higher levels of symptoms. Medium to strong positive correlations were expected between DASS21 and GPQ18, GPQ9, and GSPQ, as well as negative with GTQ.

#### General Self-Efficacy Scale

The Polish version of General Self-Efficacy Scale (GSES [24, 25]) was used to measure self-perceived self-efficacy. It comprises ten items assessed on a 4-point Likert scale (1 = *not at all true*, 4 = *exactly true*), which enable to calculate a general score (the higher the score, the higher the level of general self-efficacy). Medium negative correlations were expected between GSES and GPQ18, GPQ9, GSPQ, and positive with GTQ.

#### Acceptance and Action Questionnaire–II

The Polish version of the Acceptance and Action Questionnaire-II (AAQ-II [26, 27]) was used to measure psychological inflexibility. It consists of seven statements. Participants rate each statement on a 7-point scale, ranging from 1 = *never true* to 7 = *always true*. Higher scores indicate higher psychological inflexibility. Medium positive correlations were expected between AAQ-II and GPQ18, GPQ9, GSPQ, and negative with GTQ.

#### Cognitive Fusion Questionnaire

The Polish version of Cognitive Fusion Questionnaire (CFQ [28, 29]) was used to measure the level of cognitive fusion. The CFQ consists of seven items with a 7-point Likert-type scale (7 = *always true*; 1 = *never true*). Medium to strong positive correlations were expected between the CFQ and GPQ18, GPQ9, GSPQ, and negative–with GTQ.

#### Valuing Questionnaire

The Polish version of Valuing Questionnaire (VQ [28, 30]) was used to measure the general valued living during the past week. VQ consists of ten items and a 6-point Likert scale (6 = *completely true*; 0 = *not at all true*). It contains two subscales: Progress (defined as enactment of values, including clear awareness of what is personally important, and perseverance), as well as Obstruction (defined as disruption of valued living due to avoidance of unwanted experience and distraction from values). It was expected that the VQ Progress scale will be negatively correlated with GPQ18, GPQ9, GSPQ, and positively with GTQ. Positive correlations were expected between VQ Obstruction scale and GPQ18, GPQ9, GSPQ, and negative with GTQ.

#### Rumination—Reflection Questionnaire

The Polish version of Rumination—Reflection Questionnaire– 12 (RRQ12 [31, 32]) was used to measure the level of focus on one’s own experiences (rumination) motivated by fear, and the involvement in getting to know oneself (reflection) motivated by curiosity. RRQ12 consists of twelve items assessed with a 5-point Likert scale (1 –*I strongly disagree*, 5 –*I strongly agree*) and contains two subscales–Rumination and Reflection. It was expected that RRQ Rumination scale would be positively correlated with GPQ18, GPQ9, GSPQ, and negatively with GTQ. Negative correlations were expected between RRQ Reflection scale and GPQ18, GPQ9, GSPQ, and positive with GTQ.

The reliability coefficients (Cronbach *Alphas*) of the scales were satisfactory and are presented in Table 3.

### Statistical analyses

There were no missing values within GPQ18, GTQ and GSPQ items in both samples. A cross-validation procedure was applied with the analyses done on a data from a students’ sample A and then replicated in a general sample B. Exploratory factor analysis (EFA) was conducted only on sample A and confirmatory factor analysis (CFA) only on sample B. The measurement invariance across samples (A and B) and gender was checked. Then, the corrected item-total correlations and *Cronbach Alpha* coefficients were calculated in two samples respectively. Finally, to provide information about the validity of the scales, *r*-Pearson correlations between GPQ18, GPQ9, GTQ, GSPQ and other measures were calculated (in two samples respectively). All the analyses were conducted with the use of *SPSS* v. 25, *FACTOR* v.11.05.01 [33] and *R lavaan* package [34].

## Results

### Exploratory factor analysis

The EFA was conducted with the use of *FACTOR* v. 11.05.01 based on the data from sample A. Data included in the analyses was categorical and according to Mardia’s test it did not meet the assumptions of the multivariate normal distribution, due to the exceeded kurtosis values (The results of Mardia’s test for the GPQ18 items: b = 31.68, Z(1140) = 23.81, p = 1.00 for skewness and b = 445.22, Z = 33.72, p < .001 for kurtosis. For the GPQ9 items: b = 5.10, Z(165) = 23.81, p = 1.00 for skewness and b = 118.02, Z = 14.35, p < .001 for kurtosis. For the GTQ items: b = 13.48, Z(286) = 1013.46, p = 1.00 for skewness and b = 202.88, Z = 37.59, p < .001 for kurtosis. For the GSPQ items: b = 10.90, Z(364) = 818.99.46, p = 1.00 for skewness and b = 208.94, Z = 23.71, p < .001 for kurtosis). Data was analyzed with the use of robust diagonally weighted least squares (RDWLS) extraction method with polychoric correlations and robust Promin rotation [33]. The number of dimensions was determined by means of the optimal implementation of parallel analysis (PA [35]). The Unidimensional Congruence (UniCo), Explained Common Variance (ECV), and Mean of Item Residual Absolute Loadings (MIREAL) indexes were used to assess the unidimensionality. Values larger than .95 and .85 in UniCo and ECV, respectively, as well as a value lower than .30 for the MIREAL suggest that data can be treated as essentially unidimensional [36]. In all the analyses the 95% bootstrap confidence intervals were estimated based on 500 samples.

#### GPQ18

The Bartlett’s statistic was statistically significant (5102.1(153), *p* < .001), and the result of the Kaiser-Meyer-Olkin (KMO) test was satisfactory (.93, *95%CI* [.90, .93]). The PA suggested extracting one factor accounting for 55.08% of variance (eigenvalue = 9.10). Table 1 shows that factor loadings were high for all the items: from .50 (item 2) to .86 (item 13). The UniCo, ECV and MIREAL values suggest that the data of the GPQ-18 can be treated as unidimensional (UniCo = .97 (*95% CI* [.96, .99], ECV = .90, (*95%CI* [.86, .90], MIREAL = .22, *95% CI* [.19, .24]).

**Table 1:** The Polish version of GPQ18, GPQ9, GTQ and GSPQ items’ factor loadings and corrected item-total correlations.

Items	Factor 1	Corrected item-total correlation
*GPQ18*		
1. Mój nastrój zależy od tego, co myślą o mnie moi przyjaciele.	.681	.629
2. Bardzo przejmuję się tym, co myślą o mnie moi przyjaciele.	.497	.444
3. Czuję, że moja praca nie jest warta wysiłku, jeżeli inni ludzie jej nie doceniają.	.615	.569
4. To dla mnie bardzo ważne, aby czuć się akceptowanym przez innych.	.740	.661
5. Żeby czuć się szczęśliwym, potrzebuję być doceniany/a przez innych ludzi.	.782	.703
6. Moje poczucie własnej wartości zależy od tego, co inni ludzie o mnie myślą i mówią.	.844	.764
7. Moim głównym celem w życiu jest bycie rozpoznawanym i poważanym przez otaczających mnie ludzi.	.695	.657
8. Duży wpływ na moje decyzje mają opinie innych osób.	.796	.757
9. Bardzo przejmuję się tym, aby prezentować idealny obraz siebie.	.607	.560
10. To co robię nic by nie znaczyło, gdyby inni nie mogli tego zobaczyć.	.645	.586
11. Ciężka praca ma wartość tylko wtedy, gdy inni ludzie ją dostrzegają.	.627	.561
12. Jest dla mnie ważne, aby inni ludzie mieli w głowie mój pozytywny obraz.	.776	.727
13. Potrzebuję aprobaty ze strony innych osób, aby czuć się dobrze ze sobą.	.857	.796
14. Nie mogę zawieść oczekiwań innych osób wobec mnie.	.670	.631
15. Przed podjęciem decyzji potrzebuję, aby inni ludzie rozumieli moje powody.	.700	.657
16. Przy podejmowaniu decyzji bardziej doceniam rady innych niż własne zdanie.	.654	.591
17. Przed zrobieniem czegoś ważnego proszę innych o poradę.	.588	.544
18. Obawa przed krytyką powstrzymuje mnie od robienia różnych rzeczy.	.719	.674
*GPQ9*		
1. Bardzo przejmuję się tym, co myślą o mnie moi przyjaciele.	.493	.428
2. To dla mnie bardzo ważne, aby czuć się akceptowanym przez innych.	.758	.659
3. Żeby czuć się szczęśliwym, potrzebuję być doceniany/a przez innych ludzi.	.812	.715
4. Moje poczucie własnej wartości zależy od tego, co inni ludzie o mnie myślą i mówią.	.867	.758
5. Duży wpływ na moje decyzje mają opinie innych osób.	.745	.706
6. To co robię nic by nie znaczyło, gdyby inni nie mogli tego zobaczyć.	.663	.582
7. Ciężka praca ma wartość tylko wtedy, gdy inni ludzie ją dostrzegają.	.652	.560
8. Potrzebuję aprobaty ze strony innych osób, aby czuć się dobrze ze sobą.	.859	.779
9. Przy podejmowaniu decyzji bardziej doceniam rady innych niż własne zdanie.	.578	.511
*GTQ*		
1. Kiedy dostrzegam, że coś nie działa, próbuję czegoś innego.	.679	.614
2. Lubię dowiadywać się, jak coś działa i wyciągać swoje własne wnioski.	.690	.632
3. Łatwo dostosowuję się do zmian.	.668	.596
4. Potrafię znaleźć nowe rozwiązania problemów.	.778	.702
5. Podejmuję decyzje w oparciu o swoje doświadczenie, a nie o to, co mówią inni.	.692	.634
6. Lubię próbować różnych podejść, aby zobaczyć które jest lepsze.	.723	.654
7. Jestem dobry/a w znajdywaniu bardziej skutecznych sposobów wykonywania zadań.	.789	.711
8. Kiedy zauważam, że coś nie działa, szybko zmieniam swój sposób postępowania.	.745	.676
9. Z łatwością uczę się na konsekwencjach swoich działań.	.750	.682
10. Kiedy zdam sobie sprawę, że nie miałem/am racji, zmieniam swój sposób myślenia i działania.	.645	.552
11. Podejmuję decyzje na bazie uzyskanych wcześniej wyników.	.721	.621
*GSPQ*		
1. Czuję, że tracę kontrolę nad życiem, jeżeli moje osobiste sprawy nie są w idealnej równowadze.	.749	.684
2. Potrzebuję kontrolować swoje lęki, żeby nie czuć się słabym/ą.	.699	.638
3. Szukam odpowiedzi na wszystko, aby nie czuć się głupim/ą.	.678	.632
4. Denerwuje mnie, że nie mogę robić wszystkiego w ten sam sposób.	.643	.586
5. Czuję się pogubiony/a, jeżeli nie jestem w stanie wykonać zaplanowanych działań.	.816	.741
6. Muszę dobrze traktować innych ludzi, aby nie czuć się złym człowiekiem.	.576	.542
7. Jeżeli nie trzymam się mocno w jednej pozycji, czuję się słaby/a.	.562	.510
8. Potrzebuję mieć w życiu porządek, aby nie czuć, że tracę kontrolę.	.793	.706
9. Kiedy muszę spędzić dużo czasu na jednej aktywności, zarzucam sobie, że nie poświęcam czasu innym rzeczom.	.625	.581
10. Czuję zdezorientowany/a, gdy nie mogę podążać za swoją rutyną.	.731	.650
11. Aby być usatysfakcjonowanym/ą, muszę wszystko zrobić perfekcyjnie, tak jak sobie tego życzę.	.673	.599
12. Czuję się tak, jakbym gubił/a się w swoim życiu, jeżeli nie spełniam swoich własnych oczekiwań.	.705	.642

#### GPQ9

The Bartlett’s statistic was statistically significant (2375.6(36), *p* < .001), and the result of the Kaiser-Meyer-Olkin (KMO) test was satisfactory (.89, *95%CI* [.85, .90]). The PA suggested extracting one factor accounting for 59.96% of variance (eigenvalue = 4.99). Table 1 shows that factor loadings were high for all the items: from .49 (item 1) to .87 (item 4). The UniCo, ECV and MIREAL values suggest that the data of the GPQ-9 can be treated as unidimensional (UniCo = .95 (*95% CI* [.92, .98], ECV = .84, (*95%CI* [.81, .87], MIREAL = .28, *95% CI* [.24, .32]).

#### GTQ

The Bartlett’s statistic was statistically significant (2742.6(55), *p* < .001), and the result of the Kaiser-Meyer-Olkin (KMO) test was satisfactory (.92, *95%CI* [.88, .92]). The PA suggested extracting one factor accounting for 60.71% of variance (eigenvalue = 6.06). Table 1 shows that factor loadings were high for all the items: from .65 (item 10) to .79 (item 7). The UniCo, ECV and MIREAL values suggest that the data of the GTQ can be treated as unidimensional (UniCo = .98 (*95% CI* [.97, .99], ECV = .88, (*95%CI* [.86, .91], MIREAL = .22, *95% CI* [.17, .24]).

#### GSPQ

The Bartlett’s statistic was statistically significant (2762.5(66), *p* < .001), and the result of the Kaiser-Meyer-Olkin (KMO) test was satisfactory (.91, *95%CI* [.87, .92]). The PA suggested extracting one factor accounting for 59.69% of variance (eigenvalue = 6.07) 58.74% of variance (eigenvalue = 6.14). Table 1 shows that factor loadings were high for all the items: from .56 (item 7) to .82 (item 5). The UniCo, ECV and MIREAL values suggest that the data of the GSPQ can be treated as unidimensional (UniCo = .98 (*95% CI* [.98, .99], ECV = .89, (*95%CI* [.87, .91], MIREAL = .19, *95% CI* [.14, .20]).

Summarizing, the EFA results suggest that all the scales measure unidimensional latent constructs and the one-factor solutions explain a significant portion of variance in each case.

### Confirmatory factor analysis

**Fig 1:**
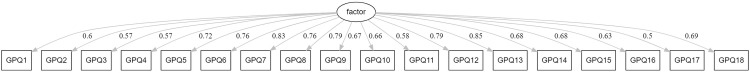
Standardized solution of the GPQ18 one-factor model in a Polish sample.

**Fig 2:**
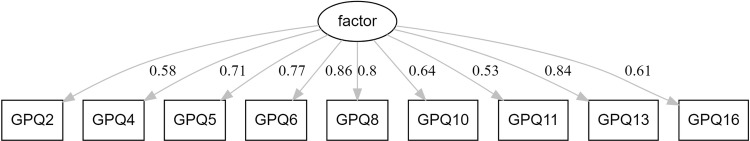
Standardized solution of the GPQ9 one-factor model in a Polish sample.

**Fig 3:**
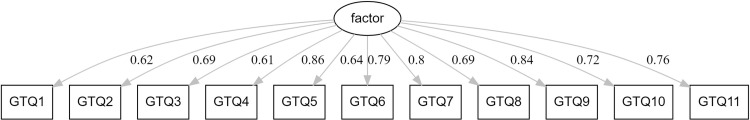
Standardized solution of the GTQ one-factor model in a Polish sample.

**Fig 4:**
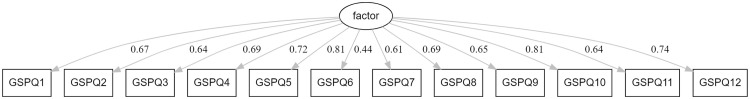
Standardized solution of the GSPQ one-factor model in a Polish sample.

The CFA was conducted with the use of the R lavaan package to analyze the fit of the one-factor model of the GPQ18, GPQ9, GTQ and GSPQ in a general sample B. A weighted least squares–mean (WLSM) estimation method with polychoric correlations was utilized. Goodness of fit was evaluated using the robust chi-square test, robust root-mean-square error of approximation (*RMSEA*), robust comparative fit index (*CFI*), the robust Tucker-Lewis index (*TLI*), and standardized root-mean-square residual (*SRMR*; the last one only in CFA). Hu and Bentler [37] proposed the following criteria of good model fit: *RMSEA*≤.10; *SRMR*≤.08; *CFI*≥.90; *TLI*≥.95, however recently the controversies to their application to categorical data have been raised [38]. Shi and Maydeu-Olivares [39] showed that *SRMR* is less sensitive to the choice of estimator, thus it is also reported. Standardized factor loading estimates are shown in Figs 1–4.

#### GPQ18

The one-factor model exhibited a non-satisfactory fit due to the high value of robust *RMSEA*: robust *χ²*(135) = 2729.54, *p* < .001; robust *RMSEA* = .108 (*95% CI*: [.105, .112]), *SRMR* = .066, robust *CFI* = .983, robust *TLI* = .980. Therefore, we decided to analyse modification indices (*MI*) with a minimum value = 10. *MI* are univariate score tests that reflect the improvement of model fit after allowing some of the parameters to be free. After careful examination of *MI* we decided to modify the model allowing the error terms between items 16 and 17 (*MI* = 97.52), 10 and 11 (*MI* = 95.12), 4 and 5 (*MI* = 91.93), as well as 1 and 2 (*MI* = 71.56) to correlate. The decision was based on the high *MI* values as well as semantic equivalence of the paired items. The constraint was released one by one and resulted in the acceptable model fit: robust *χ²*(131) = 1937.53, *p* < .001; robust *RMSEA* = .090 (*95% CI*: [.087, .094]), *SRMR* = .056, robust *CFI* = .988, robust *TLI* = .986. The same procedure was followed in the case of GPQ9 and GSPQ, which is described below.

#### GPQ9

The one-factor model exhibited a non-satisfactory fit due to the high value of robust RMSEA: robust *χ²*(27) = 810.56, *p* < .001; robust *RMSEA* = .129 (*95% CI*: [.122, .137]), *SRMR* = .064, robust *CFI* = .984, robust *TLI* = .979. Again, based on modification indices (*MI*), the error terms between items 10 and 11 (*MI* = 123.45), and 4 and 5 (*MI* = 75.76) were allowed to correlate. This improved the model fit: robust *χ²*(25) = 340.64, *p* < .001; robust *RMSEA* = .082 (*95% CI*: [.074, .90]), *SRMR* = .044, robust *CFI* = .994, robust *TLI* = .992.

#### GTQ

The one-factor model exhibited a satisfactory fit: robust *χ²*(44) = 680.31, *p* < .001; robust *RMSEA* = .089 (*95% CI*: [.083, .095]), *SRMR* = .045, robust *CFI* = .992, robust *TLI* = .991.

#### GSPQ

The one-factor model exhibited a non-satisfactory fit due to the high value of robust RMSEA: robust *χ²*(54) = 854.54, *p* < .001; robust *RMSEA* = .097 (*95% CI*: [.092, .103]), *SRMR* = .055, robust *CFI* = .985, robust *TLI* = .982. one more time, based on modification indices (*MI*), the error terms between items 1 and 2 (*MI* = 75.93), 11 and 12 (*MI* = 60.53) were allowed to correlate. This improved the model fit: robust *χ²*(52) = 553.44, *p* < .001; robust *RMSEA* = .078 (*95% CI*: [.072, .084]), *SRMR* = .048, robust *CFI* = .991, robust *TLI* = .989.

Summarizing, the results of CFA generally confirmed the one-factor solution obtained in original studies for each scale. In the case of three scales (GPQ18, GPQ9 and GSPQ) the original model presented a non-acceptable fit and some modifications were applied to obtain at least acceptable fit of the model. The final solutions present non-significant chi-square statistics (however, because of the big sample sizes this particular statistic is not a reliable source about the model fit, see [40]), satisfactory robust *CFI*, robust *TLI* and *SRMR* and at least acceptable values of robust *RMSEA*. Factor loadings were moderately or strongly related to their purported latent factor in the case of each scale.

### Measurement invariance across samples (A and B) and gender

Metric, scalar and strict invariance across both samples (A and B) and gender were conducted. The relative fits of four increasingly restrictive models were compared: the multigroup baseline model (allowing factor loadings to vary across groups while the factor structure was identical across groups (i.e., configural invariance), the metric invariance model (placing equality constraints on factor loadings across groups), the scalar invariance model (placing equality constraints on factor loadings and item intercepts), and the strict invariance model (placing equality constraints on factor loadings, item intercepts and residuals). The models were compared taking into account the differences in robust *RMSEA (ΔRMSEA)*, *CFI (ΔCFI)*, and *TLI (ΔTLI)* indexes between nested models, with *ΔCFI* being regarded as least affected by the model complexity and sample size [41]. Although the chi-square statistics and their differences between the models are also presented, due to the big sample sizes they should not be treated as decisive; a *ΔCFI*, *ΔTLI and ΔRMSEA* less than .01 indicated invariance (see [40–43]).

The results of the analyses are presented in Table 2. Baseline models present well-fit in the case of each scale. When it comes to the invariance, it can be concluded that metric, scalar and strict invariance are supported across both samples A and B in the case of each scale except the GPQ9. At the same time, the measurement can be treated as invariant regardless of the gender in the case of each scale.

**Table 2:** Measurement invariance across samples (A and B) and gender.

Model		*χ² (df)*	*χ² diff*	*p diff*	*RMSEA*	*∆RMSEA*	*CFI*	*∆CFI*		*TLI*	*∆TLI*
GPQ18	Measurement invariance across samples A and B										
	Baseline model	293.83 (262)			.044		.991			.990	
	Metric invariance	375.51 (279)	26.88	.060	.045	-.001	.990	-.001		.989	.001
	Scalar invariance	539.35 (296)	328.71	< .001	.055	-.010	.985	-.005		.984	.005
	Strict invariance	554.75 (314)	23.60	.169	.054	.001	.985	.000		.985	-.001
	Measurement invariance across gender										
	Baseline model	310.61 (262)			.046		.991			.989	
	Metric invariance	447.51 (279)	46.85	< .001	.051	-.005	.988	.003		.987	.002
	Scalar invariance	523.78 (296)	157.13	< .001	.054	-.003	.985	.003		.985	.002
	Strict invariance	545.62 (314)	34.55	.011	.053	.001	.985	.000		.985	.000
GPQ9	Measurement invariance across samples A and B										
	Baseline model	77.72 (50)			.054		.991			.986	
	Metric invariance	90.02 (58)	8.66	.372	.050	.004	.990		.001	.988	.016
	Scalar invariance	208.73 (66)	248.85	< .001	.077	-.027	.975		.015	.972	.016
	Strict invariance	213.70 (75)	8.11	.523	.072	.005	.975		.000	.976	-.004
	Measurement invariance across gender										
	Baseline model	84.76 (50)			.057		.990			.985	.002
	Metric invariance	117.92 (58)	23.18	.003	.060	-.003	.987		.003	.983	.005
	Scalar invariance	169.63 (66)	114.62	< .001	.068	-.008	.980		.007	.978	.005
	Strict invariance	180.73 (75)	19.01	.025	.065	.003	.979		.001	.980	-.002
GTQ	Measurement invariance across samples A and B										
	Baseline model	63.07 (82)			.029		.996			.995	
	Metric invariance	133.90 (92)	35.98	< .001	.042	-.013	.991		-.005	.989	.006
	Scalar invariance	169.08 (102)	66.56	< .001	.046	-.004	.988		-.003	.987	.002
	Strict invariance	181.70 (113)	17.37	.097	.045	.001	.987		-.001	.988	-.001
	Measurement invariance across gender										
	Baseline model	60.10 (82)			.029		.996			.995	
	Metric invariance	106.37 (92)	24.44	.007	.035	-.006	.994		-.002	.992	.003
	Scalar invariance	116.34 (102)	19.24	.037	.035	.000	.993		-.001	.993	-.001
	Strict invariance	133.10 (113)	24.14	.012	.035	.000	.992		-.001	.992	.001
GSPQ	Measurement invariance across samples A and B										
	Baseline model	130.56 (104)			.041		.991			.988	
	Metric invariance	185.09 (115)	26.38	.006	.045	-.004	.987		-.004	.986	.002
	Scalar invariance	235.92 (126)	90.02	< .001	.050	-.005	.983		-.004	.982	.004
	Strict invariance	240.29 (138)	6.23	.904	.047	.003	.984		.001	.984	-.002
	Measurement invariance across gender										
	Baseline model	129.82 (104)			.040		.991			.988	
	Metric invariance	158.13 (115)	13.18	.282	.039	.001	.990		-.001	.989	-.001
	Scalar invariance	186.00 (126)	48.03	< .001	.041	-.002	.988		-.002	.988	.001
	Strict invariance	193.24 (138)	10.18	.600	.039	.002	.988		.000	.989	-.001

*Note*: GPQ18 –the 18-item version of Generalized Pliance Questionnaire, GPQ9 –the 9-item version of Generalized Pliance Questionnaire, GTQ–Generalized Tracking Questionnaire, GSPQ—Generalized Self-Pliance Questionnaire.

### Validity

The bivariate *r*-Pearson correlations were calculated between GPQ18, GPQ9, GTQ and GSPQ themselves and between adapted scales and other tools measuring life satisfaction, the level of depression, anxiety and stress, the level of perceived general self-efficacy, psychological inflexibility, cognitive fusion, general valued living as well as rumination and reflection. The results are presented in Table 3. They generally support the convergent and divergent validity of adapted scales and are consistent across the samples (with differences not being tested directly).

**Table 3:** Descriptive statistics and correlations between GPQ18, GPQ9, GTQ, GSPQ and other measures’ scales in both samples.

Scales	*α*	*M* (*SD*)	Skewness (*SE*)	Kurtosis (*SE*)	GPQ18	GPQ9	GTQ	GSPQ
Sample A								
SWLS	.873	22.42 (6.09)	.23 (.12)	-.22 (.23)	-.226**	-.206**	.353**	-.231**
DASS Depression	.913	6.51 (5.38)	.74 (.12)	-.36 (.23)	.312**	.295**	-.354**	.402**
DASS Anxiety	.884	5.40 (5.01)	.84 (.12)	-.24 (.23)	.319**	.293**	-.295**	.430**
DASS Stress	.888	8.29 (5.14)	.37 (.12)	-.62 (.23)	.342**	.312**	-.302**	.468**
GSES	.886	29.92 (4.57)	-.21 (.12)	.95 (.23)	-.267**	-.240**	.620**	-.277**
AAQ—II	.933	24.09 (10.37)	.23 (.12)	-.67 (.23)	.446**	.410**	-.338**	.543**
CFQ	.954	27.62 (10.28)	-.07 (.12)	-.57 (.23)	.437**	.395**	-.263**	.567**
VQ Progress	.830	16.89 (6.12)	-.27 (.12)	-.38 (.23)	-.119*	-.113*	.432**	-.071
VQ Obstruction	.847	12.51 (6.72)	.13 (.12)	-.68 (.23)	.343**	.320**	-.296**	.436**
RRQ Rumination	.825	20.47 (5.12)	-.20 (.12)	-.27 (.23)	.422**	.397**	-.213**	.455**
RRQ Reflection	.820	22.80 (4.79)	-.27 (.12)	-.72 (.23)	.040	.029	.238**	.035
GPQ18	.934	71.12 (17.53)	.02 (.12)	.14 (.23)		.967**	-.199**	.595**
GPQ9	.884	35.45 (9.03)	-.01 (.12)	.20 (.23)			-.176**	.534**
GTQ	.903	53.47 (8.95)	.07 (.12)	.03 (.23)				-.174**
GSPQ	.903	47.90 (12.36)	-.28 (.12)	.27 (.23)				
Sample B								
SWLS	.896	19.50 (6.09)	-.34 (.10)	.07 (.19)	.008	.000	.322**	-.104**
DASS_D	.920	6.25 (5.35)	.76 (.10)	-.23 (.19)	.298**	.280**	-.185**	.440**
DASS_A	.874	4.43 (4.45)	1.13 (.10)	.80 (.19)	.279**	.261**	-.141**	.401**
DASS_S	.903	7.04 (4.95)	.48 (.10)	-.44 (.19)	.289**	.268**	-.129**	.489**
GSES	.927	29.40 (4.87)	-.70 (.10)	2.06 (.19)	-.108**	-.112**	.581**	-.105**
AAQ	.941	23.27 (10.42)	.35 (.10)	-.59 (.20)	.429**	.405**	-.234**	.523**
CFQ	.961	23.82 (10.79)	.25 (.10)	-.64 (.20)	.4398**	.361**	-.184**	.589**
VQP	.829	15.99 (6.16)	-.22 (.10)	-.11 (.20)	.042	.022	.454**	.077
VQO	.858	11.82 (6.92)	.24 (.10)	-.57 (.20)	.406**	.388**	-.220**	.527**
RRQ_RU	.844	18.61 (5.69)	-.09 (.10)	-.55 (.20)	.350**	.321**	-.217**	.508**
RRQ_RE	.694	19.31 (4.17)	.37 (.10)	.47 (.20)	.121**	.096*	.130**	.270**
GPQ18	.934	67.47 (18.05)	.04 (.09)	.11 (.19)		.974**	.007	.594**
GPQ9	.890	33.82 (9.48)	.03 (.09)	-.05 (.19)			.001	.550**
GTQ	.910	53.31 (9.39)	.11 (.09)	.08 (.19)				.067
GSPQ	.899	46.72 (12.16)	-.04 (.09)	.24 (.19)				

Note: *M*–mean, *SD*–standard deviation, *SE*–standard error, *α* –Cronbach *Alpha*, SWLS–Satisfaction with Life Scale, DASS Depression–the Depression scale of Depression, Anxiety, and Stress Scale, DASS Anxiety–the Anxiety scale of Depression, Anxiety, and Stress Scale, DASS Stress–the Stress scale of Depression, Anxiety, and Stress Scale, GSES–General Self-efficacy Scale, AAQ–II–Acceptance and Action Questionnaire–II, CFQ–Cognitive Fusion Questionnaire, VQ Progress–the Progress scale of Valuing Questionnaire, VQ Obstruction–the Obstruction scale of Valuing Questionnaire, RRQ Rumination–the Rumination scale of Rumination–Reflection Questionnaire, RRQ Reflection–the Reflection scale of Rumination–Reflection Questionnaire, GPQ18 –the 18-item version of Generalized Pliance Questionnaire, GPQ9 –the 9-item version of Generalized Pliance Questionnaire, GTQ–Generalized Tracking Questionnaire, GSPQ—Generalized Self-Pliance Questionnaire;* *p* < .05, ** *p* < .01

The correlation between GPQ18 and GPQ9 were very high in both samples, suggesting that both tools measure the same construct (accordingly with the expectations). Pliance (measured by both GPQ18 and GPQ9) was strongly related to self-pliance, which was also consistent with a priori hypotheses. Tracking was weakly (and negatively) or non-significantly related to pliance and self-pliance, showing that these constructs reflect different and strongly independent rule-governed behaviors.

Pliance (as measured by GPQ18 and GPQ9) and self-pliance was moderately or strongly and positively related to the level of depressive, anxiety and stress symptoms, psychological inflexibility, cognitive fusion, obstruction of valued living and rumination. They showed weak positive or non-significant relationships with reflection, and progress in valued living. The relationship with general self-efficacy and life satisfaction was weak and negative or non-significant. These least results seem less expected, suggesting that possibly life satisfaction and self-efficacy are less affected by the level of presented pliance and self-pliance (the hypothesis worth further testing).

Tracking showed to be positively and moderately or strongly related to life satisfaction, general self-efficacy and progress in valued living and weakly with reflection. It was also negatively related (at least moderately) with depressive, anxiety and stress symptoms, psychological inflexibility, cognitive fusion, obstruction of valued living and rumination. The results are generally consistent with set hypotheses and those obtained in a study of original scale [12].

## Discussion

The aim of the study was to evaluate the psychometric properties of Polish adaptations of the questionnaires measuring rule-governed behaviors such as pliance, self-pliance and tracking.

The EFA and CFA analyses corroborated unidimensional structure with high factor loadings found in the original validation studies for each of the measurements: GTQ [12], GSPQ [10] and both versions of GPQ: GPQ18 and GPQ9 [11]. Model fit to data was acceptable. All of the adapted scales presented good reliability (internal consistency) and item-total correlations.

Polish versions of questionnaires presented significant correlations in the expected directions with relevant psychological variables in line with the original studies [10–12]. Regarding ACT processes, pliance (measured by Polish versions of GPQ9 and GPQ18) and self-pliance (GSPQ) was positively related to, cognitive fusion and obstruction of valued living, and not related or negatively correlated with progress in valued living. This last score is slightly different from the results obtained by Ruiz and colleagues [11] and by Ruiz and Suárez-Falcón and colleagues [10], who showed consistent negative correlations between GPQ9, GPQ18 and GSPQ and progress in valued living. In our study, only in the student sample GPQ18 and GPQ9 were negatively correlated with progress in valued living, whereas in the general sample, as well as for GSPQ there were no significant correlations.

Pliance and self-pliance were negatively related to psychological flexibility as measured by AAQ-II [26, 27] similarly to the results obtained by Ruiz and Suárez-Falcón and colleagues [10] and Ruiz and colleagues [11] who also used AAQ-II in their studies. Despite AAQ-II have been questioned as a precise and adequate measure of psychological flexibility [44, 45], the result is in line with the results obtained by researchers that used different measurements with greater construct validity (e.g. CompACT [46]). Pliance and self-pliance were positively related with rumination and emotional symptoms in line with the original validation studies [10, 11] and showed weak and positive or non-significant relationship with reflection. Finally, they presented weak and negative or non-significant correlations with life satisfaction and self-efficacy. In contrast to the original studies, GPQ18 and GPQ9 in the general sample were not related to life satisfaction.

Finally pliance, self-pliance were positively correlated in both samples, yet pliance and self-pliance and tracking were negatively correlated only in the student sample, which is contrary to expectations and needs further replication.

Summarizing, pliance and self-pliance seem negatively related to psychological flexibility. Although the results of original studies suggest pliance may be a process leading to lower psychological flexibility and the lower level of life satisfaction, the latter was not found in the Polish sample.

Tracking, as measured by the Polish version of GTQ, was positively related to progress in valued living and negatively related with psychological inflexibility, cognitive fusion, obstruction to valued living. Moreover, it was positively correlated with life satisfaction, general self-efficacy and reflection and negatively with rumination and emotional symptoms in line with the original validation study [12]. The results support the idea that tracking, i.e. being in direct contact with contingencies and following the real consequences of behavior, is a process which may support living a valuable life, the feeling of greater self-efficacy, which may lead to greater satisfaction with life and, possibly, general health.

Despite indicating the validity of the Polish adaptations of GPQ, GSPQ and GTQ, the present study should be complemented by further research. Although in general correlations with other measures are in line with original validation studies, there may be questions regarding lack or very weak correlation of pliance with life satisfaction. At the same time, the relationship between tracking and life satisfaction as well as self-efficacy was consistent in the original and Polish study. The explanation may be the cultural differences between Polish and South American societies. The main difference between pliance and tracking is apparent source of reinforcement for rule-following: in pliance it is social or arbitrary, in tracking–non arbitrary. For some reason following the behavior considered as socially approved in Poland seems to have little impact on life satisfaction while it may still have detrimental effects for individuals’ general functioning (because of negative relationship with psychological flexibility). Differences between Irish as well as Columbian adolescents in pliance and psychological inflexibility has been recently reported by Stapleton et al. [47]. Further longitudinal research allowing for cause-effect conclusions should determine the relationship between pliance, tracking, life satisfaction and self-efficacy in cross-cultural studies.

The present study has some more limitations. First of all, the validity of the measurements should be tested in clinical samples due to its potential utility in research and practice in the area of psychopathology. Secondly, the validity of GTQ should also be confirmed in future studies including its relationship with executive functions. Further studies should include more precise and adequate measurements of psychological flexibility. Furthermore, the stability of the results needs to be established in additional study. The study has been conducted online and we have not compared the mode of administration of measurements (e.g. online vs pen-and-paper methods might be regarded by participants as providing different levels of anonymity, and this may lead to differences in how socially biased responding would be [48]). Finally, the samples were not representative of the Polish general and student’s population.

In conclusion, this study contributes with the GPQ, GSPQ and GTQ to the Polish language and they appear to be adequate to be used in Polish samples which may accelerate the development of research on pliance, self-pliance and tracking in Polish language and support clinicians working with clients.
